# Biological Effects of Glucosinolate Degradation Products from Horseradish: A Horse that Wins the Race

**DOI:** 10.3390/biom10020343

**Published:** 2020-02-21

**Authors:** Marijana Popović, Ana Maravić, Vedrana Čikeš Čulić, Azra Đulović, Franko Burčul, Ivica Blažević

**Affiliations:** 1 Department of Organic Chemistry, Faculty of Chemistry and Technology, University of Split, Ruđera Boškovića 35, 21000 Split, Croatia; azra@ktf-split.hr; 2 Department of Biology, Faculty of Science, University of Split, Ruđera Boškovića 33, 21000 Split, Croatia; amaravic@pmfst.hr; 3 Department of Medical Chemistry and Biochemistry, School of Medicine, University of Split, Šoltanska 2, 2100 Split, Croatia; vcikesc@mefst.hr; 4 Department of Analytical Chemistry, Faculty of Chemistry and Technology, University of Split, Ruđera Boškovića 35, 21000 Split, Croatia; franko@ktf-split.hr

**Keywords:** horseradish, glucosinolates, isothiocyanates, microwave-assisted isolation, antimicrobial activity, cytotoxic activity

## Abstract

Horseradish degradation products, mainly isothiocyanates (ITC) and nitriles, along with their precursors glucosinolates, were characterized by GC-MS and UHPLC-MS/MS, respectively. Volatiles from horseradish leaves and roots were isolated using microwave assisted-distillation (MAD), microwave hydrodiffusion and gravity (MHG) and hydrodistillation (HD). Allyl ITC was predominant in the leaves regardless of the isolation method while MAD, MHG, and HD of the roots resulted in different yields of allyl ITC, 2-phenylethyl ITC, and their nitriles. The antimicrobial potential of roots volatiles and their main compounds was assessed against sixteen emerging food spoilage and opportunistic pathogens. The MHG isolate was the most active, inhibiting bacteria at minimal inhibitory concentrations (MICs) from only 3.75 to 30 µg/mL, and fungi at MIC_50_ between <0.12 and 0.47 µg/mL. Cytotoxic activity of volatile isolates and their main compounds were tested against two human cancer cell lines using MTT assay after 72 h. The roots volatiles showed best cytotoxic activity (HD; IC_50_ = 2.62 μg/mL) against human lung A549 and human bladder T24 cancer cell lines (HD; IC_50_ = 0.57 μg/mL). Generally, 2-phenylethyl ITC, which was tested for its antimicrobial and cytotoxic activities along with two other major components allyl ITC and 3-phenylpropanenitrile, showed the best biological activities.

## 1. Introduction

Food and pharmaceutical industries exert significant effort in exploring natural metabolites as novel solutions for the improvement of food safety, as well as for introduction of alternative regimens in the treatment of various infections [1,2]. This, however, represents a serious challenge regarding the global problem of dissemination of bacterial resistance to antibiotics, with ESKAPE group of pathogens (*Enterococcus faecium*, *Staphylococcus aureus*, *Klebsiella pneumoniae*, *Acinetobacter baumannii*, *Pseudomonas aeruginosa*, and *Enterobacter* species) being responsible for most nosocomial infections, and successfully “escaping” the effect of commercial antimicrobial drugs [3].

Glucosinolates (GSLs) are major constituents of horseradish (*Armoracia rusticana* P.Gaertn., B.Mey. & Scherb.), a large-leaved, hardy perennial plant of the Brassicaceae family [4,5]. Degradation of GSLs generates different chemical structures: isothiocyanates (ITCs), thiocyanates, nitriles, epithionitriles, and oxazolidinethiones [6,7]. GSL degradation may be initiated enzymatically, thermally, and chemically [8]. Horseradish was studied by many authors and sinigrin (1) was found as the most dominant GSL in all parts of the plant, followed by gluconasturtiin (5) and glucobrassicin (6) [9,10,11,12,13], while the major volatiles were allyl- and 2-phenylethyl ITCs [14,15,16].

ITCs represent naturally occurring bioactive compounds with various known biological activities [17]. They were previously found to exhibit broad-spectrum antibacterial potential [18,19], even against multidrug-resistant clinical strains [20]. The latter study suggests ITCs as a potential phytotherapeutic alternative to conventional antibiotics in the treatment of common infections, like those of the urinary tract caused by antibiotic-resistant *E. coli* [20]. Regular consumption of *Brassica* vegetables has also been associated with the reduced risk of cancer [21]. Chemopreventive actions of ITCs are attributed to their ability to modulate different cellular mechanisms such as: phase I and phase II drug metabolism enzymes, cell cycle arrest, apoptosis and differentiation, antioxidant and detoxication proteins, as well as proinflammatory and procarcinogen factors [22]. Park et al. showed that volatiles obtained by steam distillation of horseradish roots have good insecticidal activity [23]. Good anticandidal and antimicrobial activity of horseradish volatiles obtained with hydrodistillation was revealed in the study by Petrović et al. [15]. Dekić et al. confirmed those findings, shedding a new light on good cytotoxic and spasmolytic activities of horseradish volatiles obtained by autolysis [16]. Microwave hydrodiffusion and gravity (MHG) and microwave-assisted distillation (MAD) are new, green extraction and distillation techniques that use microwave irradiation for evenly heating the in-situ water of the plant material, inducing thermal degradation of GSLs [24,25,26]. MHG and MAD can result in different chemical profiles or different yields compared to the conventional distillation and extraction techniques [26,27].

The aim of this study was to evaluate the influence of different heating treatments on the GSLs present in the horseradish leaves and roots during microwave irradiation (using MAD and MHG techniques) and hydrodistillation (using Clevenger type apparatus, HD). GSLs were identified and quantified by their desulfo counterparts using UHPLC-DAD-MS/MS, while the volatiles produced after their thermal degradation were analyzed using GC-MS. In order to expand the knowledge of beneficial effects of horseradish volatiles, biological activities of the volatile isolates and their main compounds were investigated. Additionally, mixtures containing different ratios of the main compounds were used to reveal their possible synergistic or antagonistic effects. The antimicrobial effectiveness against a range of emerging food spoilage and opportunistic pathogens, among which clinically isolated multidrug-resistant ESKAPE strains, was assessed by the microdilution assay. Furthermore, the cytotoxic activity of horseradish volatiles and their main compounds was investigated using the MTT method against human lung (A549) and human bladder (T24) cancer cell lines.

## 2. Materials and Methods

### 2.1. Plant Material and Standards

Horseradish (leaves and roots) was collected in March 2019 from the wild-growing population in Konavle (near Dubrovnik, Croatia; 42°34′53″ N 18°13′03″ E) during the spring flowering phenological stage. The voucher specimen (ZOKAR001) is deposited at the Department of Organic Chemistry, Faculty of Chemistry and Technology, Split, Croatia. 2-Phenylethyl isothiocyanate (PEITC), 3-phenylpropanenitrile (PPCN), and allyl isothiocyanate (AITC) were purchased from Sigma Aldrich (St. Louis, MO, USA).

### 2.2. Analysis of Glucosinolates and Volatiles

#### 2.2.1. Isolation of Desulfoglucosinolates

Isolation of desulfoglucosinolates (dGSLs) from 100 mg of dried horseradish roots and leaves was performed as reported previously [28,29]. Plant material was firstly subjected to extraction in MetOH/H_2_O (70:30 *v/v*; Gram-Mol d.o.o, Zagreb, Croatia). The supernatant was loaded on mini-column filled with DEAE-Sephadex A-25 anion-exchange resin (Sigma-Aldrich, St. Louis, MO, USA) and the columns were then washed to remove the remaining non-polar compounds. To create optimal conditions for the sulfatase reaction, mini columns were washed with 20 mM NaOAc buffer (Merck, Darmstadt, Germany) followed by the addition of sulfatase (type H-1 from *Helix pomatia*; Sigma-Aldrich, St. Louis, MO, USA). The reaction was left overnight and the dGSLs were eluted the next day with ultrapure H_2_O (Merck Millipore, Burlington, MA, USA).

#### 2.2.2. HPLC-DAD Analysis of Desulfoglucosinolates

dGSLs were analyzed by high-performance liquid chromatography (Hewlett Packard 1090 Series II UV-visible, Palo Alto, CA, USA) with binary gradient solvent delivery system, autoinjector, diode-array detector (wavelength range 190–600 nm), and Nucleosil C-18 column (250 mm × 4 mm, 5 µm particle size, Macherey-Nagel GmbH & Co. KG, Düren, Germany). The flow rate of 0.8 mL/min was applied for solvent A (H_2_O) and solvent B (acetonitrile:H_2_O = 30:70 *v/v*) as followed: 0.5 min 96% A and 4% B, 28 min 14% A and 86% B, 4.0 min 14% A and 86% B, 2.0 min 5% A and 95% B, 13.0 min 5% A and 95% B, 1.0 min 96% A and 4% B, and 8.0 min 96% A and 4% B. After each run the system was allowed to equilibrate for 2 min. The column was set at room temperature (25 °C). The signals were recorded at the 227 nm by DAD detector. Quantification of dGSLs was performed using an external calibration curve of pure desulfosinigrin (range from 13.56–542.50 µM). For each individual dGSL response factors (RPF) was taken in accordance to the literature: RPF 1.0 for 1, 2 and 5 [30]; RPF 1.15 for 3, and 0.29 for 6 [31]; arbitrary 1.0 for 4.

#### 2.2.3. UHPLC-MS/MS Analysis of Desulfoglucosinolates

The analysis was performed on UHPLC-DAD-MS/MS (Ultimate 3000RS with TSQ Quantis MS/MS detector, Thermo Fischer Scientific, Waltham, MA, USA) using Hypersil GOLD column (3.0 µm, 3.0 × 100 mm, Thermo Fischer Scientific, USA). A gradient consisting of solvent A (50 μM NaCl in H_2_O) and solvent B (acetonitrile:H_2_O 30:70 *v/v*) was applied at a flow rate of 0.5 mL/min as follows: 0.14 min 96% A and 4% B; 7.84 min 14% A and 86% B; 8.96 min 14% A and 86% B; 9.52 min 5% A and 95% B; 13.16 min 5% A and 95% B; 13.44 min 96% A and 4% B; 15.68 min 96% A and 4% B. The column temperature was held at 40 °C and the injection volume was 5 µL. The system was operated in the positive ion electrospray mode and the electrospray interface was H-ESI operating with a capillary voltage of 3.5 kV at 350 °C.

#### 2.2.4. Isolation of Volatiles

Microwave-assisted distillation was performed in the Ethos X microwave system (Milestone, Brøndby, Denmark) for 30 min at 500 W. The plant material (287 g of the leaves and 374 g of the roots) was partially immersed in water and transferred into a flask inside the microwave oven. Microwaves heated the in situ water until the cell walls ruptured and the molecules of interest were released. The vapors carried the volatile compounds through the coolant into the pentane trap, which retains volatiles.

Microwave hydrodiffusion and gravity was also performed in the Ethos X microwave system for 10 min at 500 W. The plant material (516 g of the leaves and 493 g of the roots) was placed into a microwave reactor without any solvent addition. The extracts passed through a cooler before reaching the collection beaker. The volatiles from the aqueous solution were extracted with dichloromethane (T.T.T. d.o.o., Sveta Nedelja, Croatia).

Hydrodistillation (HD) of plant material (75.49 g of the leaves and 377 g of the roots) was performed in the Clevenger apparatus (DeottoLab, Zagreb, Croatia) for 2.5 h, as reported previously [32].

#### 2.2.5. GC-MS Analysis of Volatiles

Identification of volatiles from all the isolates was performed by GC-MS as previously described on both non-polar VF-5MS and polar CP Wax 52 columns (Varian Inc., Lake Forest, CA, USA) with slight modifications [33]. Briefly, the chromatographic conditions for both columns were as follows: helium as carrier gas with the flow rate 1 mL/min^−1^; injector temperature 250 °C; volume injected 1 μL and the split ratio 1:50. For the VF-5MS column temperature was programmed at 60 °C isothermal for 3 min, increased to 246 °C at a rate of 3 °C min^−1^ and held isothermal for 25 min, while for CP Wax 52 column the temperature was 70 °C isothermal for 5 min, then increased to 240 °C at a rate of 3 °C/min^−1^ and held isothermal for 25 min. For the MS, ionization voltage was set to 70 eV, ion source temperature at 200 °C and the mass scan range was 40–350 mass units. The individual peaks were identified by comparison of their retention indices and by computer matching of mass spectra against the Wiley 7-spectra library database, along with the comparison of mass spectra from the literature data [34,35,36]. All the analyses were run in duplicate and the mean values of component percentage were obtained from both columns.

### 2.3. Antimicrobial Activity

#### 2.3.1. Bacterial Strains

The antimicrobial effect of horseradish roots MHG, MAD, HD, and their main volatiles, among them PEITC and PPCN, was assessed on sixteen strains of opportunistic pathogens, including the emerging food spoilage microorganisms as well as clinical multidrug-resistant ESKAPE bacterial strains. Susceptibility testing was carried on Gram-positive *Listeria monocytogenes* ATCC 19111, *Staphylococcus aureus* (ATCC 29213 and a methicillin-resistant *S. aureus* clinical strain MRSA-1), *Enterococcus faecalis* ATCC 29212, *Streptococcus pyogenes* ATCC 19612, and *Bacillus cereus* food isolate. Gram-negative strains included *Salmonella enterica* serovar Typhimurium WDCM 00031, *E. coli* (ATCC 25922 and an extended-spectrum-beta-lactamase (ESBL)-producing multiple-resistant clinical strain), *Klebsiella pneumoniae* (ATCC 13883 and ESBL-producing multiple-resistant clinical strain), and *Acinetobacter baumannii* (ATCC 19606 and a metallo-beta-lactamase-producing multiple-resistant hospital strain). Multidrug-resistant clinical strains were obtained from the University Hospital Centre Split, Croatia. Their origin and antibiotic resistance phenotypes were described previously [37]. Antifungal activity was estimated against an environmental isolate of opportunistic pathogenic yeast *Candida albicans* and food isolates of food spoilage moulds *Penicillium citrinum* and *Aspergillus niger*.

Antibiotic susceptibility testing was carried out using Etest (AB Biodisk, Solna, Sweden) and VITEK 2 system (bioMérieux, Craponne, France). Microorganisms were stored at −80 °C and subcultured on tryptic soy agar (TSA, Biolife, Milan, Italy) or Sabouraud dextrose agar (SDA, Biolife, Milan, Italy) before the testing.

#### 2.3.2. Microdilution Assays

The antimicrobial activity was tested using a two-fold broth microdilution assay according to the guidelines of the Clinical Laboratory Standards Institute [38,39] and the protocol was described in detail previously [40]. Experiments were carried out in 96-well microtiter plates with serial two-fold dilutions of horseradish roots extract, distillates, PEITC, and PPCN as follows: hydrodistillate (4 mg/mL) was tested in a range from 800 to 0.78 µg/mL; MAD (2 mg/mL) in a range from 400 to 0.39 µg/mL; MHG (600 µg/mL) in a range from 120 to 0.12 µg/mL; PEITC and PPCN (10 mg/mL) in a range from 2000 to 1.95 µg/mL, respectively. AITC susceptibility was previously tested [40]. The mixtures of three predominant volatiles in horseradish, PEITC, PPCN, and AITC, mixed in different proportions were also included in testing in order to evaluate synergistic effects. These three main volatiles (10 mg/mL) were mixed in ratio, 7:2:1, 4:4:2, and 3:2:5, respectively. For easier comparison, the mixed solutions were then diluted to reach concentrations used for testing as previously noted. The MIC value was recorded as the lowest concentration showing no visually detectable bacterial growth in the wells and was the consensus value of the experiment performed in triplicate. For the determination of minimal bactericidal concentration (MBC), bacteria from the wells corresponding to the MIC, 2 × MIC and 4 × MIC were plated, and the MBC was recorded as the lowest concentration causing ~99.9% killing of the start inoculum.

### 2.4. Cytotoxic Activity

In order to determine cytotoxic activity of horseradish volatiles (PEITC, PPCN, AITC), individual compounds and their 7:2:1 mixture, cell viability assay (3-(4,5-dimethylthiazol-2-yl)-2,5-diphenyltetrazolium bromide, MTT) was performed as previously described [40]. Human bladder cancer cell line T24 and human lung cancer cell line A549 (LGC Standards) were incubated overnight in 96-well plates at a density of 5000 cells/well followed by incubation with test substances (in triplicate) at concentrations of 1, 5, 10, 50, and 100 µg/mL for 72 h. The cells were then incubated with 0.5 g MTT/L at 37 °C for 2 h, followed by the removal of the medium and addition of 10% dimethylsulfoxide (DMSO) for another 10 min at 37 °C. Formazan formation, the indicator of metabolically active cells, was measured at 570 nm on the microplate reader (BioSan, Riga, Latvia). For statistical analyses t-test with unequal variances was performed using statistical software GraphPad Prism 7.0 (San Diego, CA, USA) with the significance set at *p* < 0.05. The criteria used to categorize the activity against the tested cell lines was based on IC_50_ values as follows: <20 μg/mL = highly active, 21–200 μg/mL = moderately active, 201–500 μg/mL = weakly active, and >501 μg/mL = inactive [41]. The calculation of IC_50_ values was performed with the GraphPad Prism software version 7.0 (San Diego, CA, USA), normalizing the data by three independent measurements of untreated controls. The combination index value (CI) was calculated in order to quantify interactions between the compounds in a mixture as synergism (CI < 1) or antagonism (CI > 1) using a median-effect analysis by CompuSyn software [42,43].

## 3. Results and Discussion

### 3.1. Chemical Characterization

**Figure 1:**
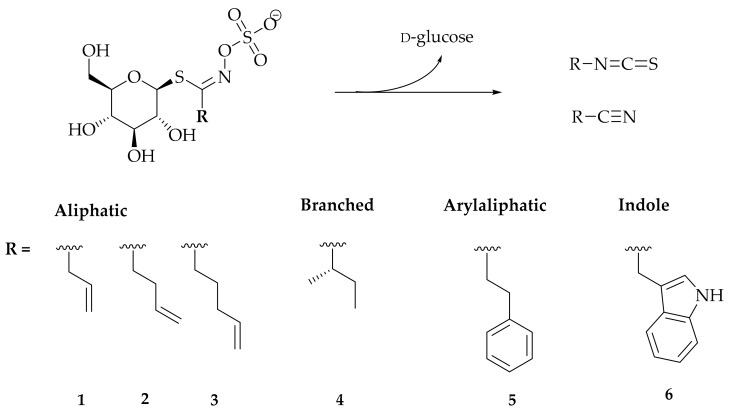
General scheme of glucosinolate degradation with the structures identified in horseradish.

Horseradish leaves and roots, collected from a wild-growing population, were analyzed qualitatively and quantitatively for the presence of GSLs by their desulfo counterparts using HPLC-DAD and UHPLC-MS/MS. The results are given in Table 1 and Figures S1 and S2 (Supplementary Materials). Generally, six GSLs were identified by UV spectra, retention time (*t*_R_) and mass spectra with commercial standards and the literature. All the structures are given in Figure 1.

The most abundant GSL in the roots was gluconasturtiin (5; 64.9%), followed by sinigrin (1; 33.91%) and glucobrassicin (6; 1.19%), while in the leaves only 1 was quantified (>99.9%). Next to 1, two other Met derived GSLs, found in traces in all part plant parts, were gluconapin (2) and glucobrassicanapin (3). Glucocochlearin (4), the only branched GSL, derived from Ile, was also detected in traces in all plant parts.

The volatiles from horseradish leaves and roots were isolated by HD, MHG, and MAD. The profiles and yields of the obtained volatiles are shown in Table 2. Regardless of the isolation method or part of the plant, the main volatiles found in each sample originated from the degradation of 1 and 5 which were the major GSLs (Table 1). Allyl ITC (AITC), but-3-enenitrile and allyl thiocyanate were identified as degradation products of 1, while 2-phenylethyl ITC (PEITC) and 3-phenylpropanenitrile (PPCN) were identified as degradation products of 5. Other volatiles, namely but-3-enyl-, pent-4-enyl- and *sec*-butyl ITC, which originated from the degradation of 2, 3, and 4, respectively were also detected in traces. Degradation volatiles of 6 were not found due to their thermal instability during GC-MS analysis.

The major GSL in the leaves 1 degraded mostly into AITC by HD (100 °C, 150 min), having 73.45%. Volatile extracts obtained by microwave-assisted techniques (MAD and MHG; 500 W, 30 min, 98 °C and 500 W, 10 min, 98 °C, respectively) also contained AITC in high percentages, i.e., 54.77 and 52.36%, but these conditions favored degradation to but-3-enenitrile having ca 37% in both isolates in comparison to HD. The major GSL in the roots 5 degraded mostly into PEITC by HD and MAD having 27.61% and 62.82%, respectively, but also having respectable amounts of PPCN i.e., 15.44% and 18.61%, respectively. On the other hand, MHG contained more PPCN (34.44%) than PEITC (30.53%).

The main volatiles in the leaves and the roots obtained by HD were ITCs (75.77% and 81.09%, respectively). The main volatiles by MAD of the leaves and the roots were ITCs as well (77.58% and 57.94%, respectively), but also contained respectable amounts of nitriles (19.66 and 37.65%, respectively); MHG of the leaves contained similar content of ITCs and nitriles as MAD, while MHG of the roots had nitrile content equal to ITCs (ca 45%). Generally, HD seemed to favor degradation to ITCs, while during microwave-assisted isolation GSLs degraded towards nitriles as well. In the conventional method, heat is transferred from the heating medium to the interior of the sample (conductive heating). On the other hand, microwave dielectric heating uses the ability of water to transform irradiation of electromagnetic waves to heat. The influence of microwave irradiation that is involved during the process of thermal degradation of GSLs can be responsible for the observed higher nitriles content in the isolates obtained by microwave-assisted isolation methods. The yields of the volatiles (Table 2) obtained by MAD and MHG were much lower than the yields obtained by HD. The volatiles isolated with MAD from the leaves were 40-fold less than isolated by HD, while MAD of horseradish roots resulted in half of the HD yield. The volatiles of the leaves and the roots had the lowest yields with the MHG.

### 3.2. Biological Activities

#### 3.2.1. Antimicrobial Effect of Horseradish and Its Main Volatiles

In order to assess the antimicrobial potential of horseradish, root volatiles obtained by HD, MAD, and MHG, three main constituents (PEITC, PPCN, and AITC), as well as their mixtures in different ratios were tested using microdilution assay on a range of spoilage and foodborne microorganisms. The results are given in Table 3 and Table 4. In our previous study, antimicrobial effects of *L. latifolium* volatile isolates, as well as their main constituent AITC, were tested against a range of spoilage and foodborne microorganisms [40].

The root MHG (mixed nitrile—ITC type constituents) extract proved to be the most active in comparison to the tested distillates i.e., HD and MAD (ITC type constituents). MHG inhibited both laboratory and clinical antibiotic-resistant bacterial strains at very low MIC values ranging from 3.75 to 30 µg/mL. The fungi were inhibited at even lower doses of MIC_50_ between <0.12 and 0.47 µg/mL. HD and MAD exhibited weaker antimicrobial activity, with MICs ranging from 25 to 100 µg/mL. Both distillates demonstrated a strong antifungal effect with MIC_50_ recorded between <0.78 and 3.125 µg/mL (Table 3). Interestingly, the lowest MICs of MHG and MAD were recorded against clinical methicillin-resistant *S. aureus* (MRSA strain) at 3.75 and 12.5 µg/mL, while *S. aureus* ATCC 29213 was susceptible at 30 and 25 µg/mL. The second most susceptible species was the important foodborne pathogen *L. monocytogenes* that was successfully inhibited at 7.5 µg/mL of MHG and 25 µg/mL of MAD. Importantly, the MIC values obtained for clinical strains were mostly very similar to those of ATCC strains, thus demonstrating the potency of horseradish volatiles even against antibiotic-resistant pathogens. Of note, when comparing sensitivity results with those obtained by the other authors, significantly better activity of HD was demonstrated against *K. pneumoniae* ATCC 13883 and *E. faecalis* ATCC 29212 strains than Petrović et al. [15]. On the other hand, excellent antifungal activity of horseradish volatiles showed herein is in concordance with a previous study [44].

In addition, the results of antimicrobial testing of main volatiles from horseradish obtained in this study show the species-specific activity of PEITC and PPCN, with MIC ranging from 15.6 to 500 µg/mL (Table 4). PEITC was more active, showing the best inhibition against MRSA and *E. coli* ATCC 25922 (15.6 µg/mL). Overall, we found no straightforward differentiation in susceptibility of Gram-negative and Gram-positive species towards these compounds, which is in agreement with previous studies on ITCs antibacterial activity [19,40].

Interesting results were obtained by testing the antimicrobial activity of PEITC, PPCN, and AITC mixtures in various ratios (Ψ_PEITC:PPCN:AITC_, Table 4). The reduction of MIC values was recorded in the case of all bacteria when comparing the activities of a specific compound to the mixtures with the other two compounds. For instance, AITC, PEITC and PPCN inhibited *A. baumannii* ATCC 19606 at 125, 125 and 250 µg/mL ([40]; Table 4) while a combination of compounds at Ψ_4:4:2_ ratio, inhibited the same strain at MIC of only 7.5 µg/mL. Moreover, clinical *A. baumannii* was also inhibited at the same Ψ_4:4:2_ ratio concentration, while the MIC values of each individual compound were 31.25, 125, and 125 µg/mL [40]. These results indicate the potential synergetic antimicrobial effect of three main volatiles from horseradish.

Moreover, the differences observed between MICs and MBCs values for extract, distillates and volatiles tested individually, particularly against Gram-positive strains favor the conclusion above. Namely, most of MBC values are 4-fold higher than MIC, which is a direct consequence of the bacteriostatic effect of ITCs as previously evidenced on *S. aureus* ATCC 29213 [40].

#### 3.2.2. Cytotoxic Activity of Horseradish and Its Main Volatiles

**Figure 2:**
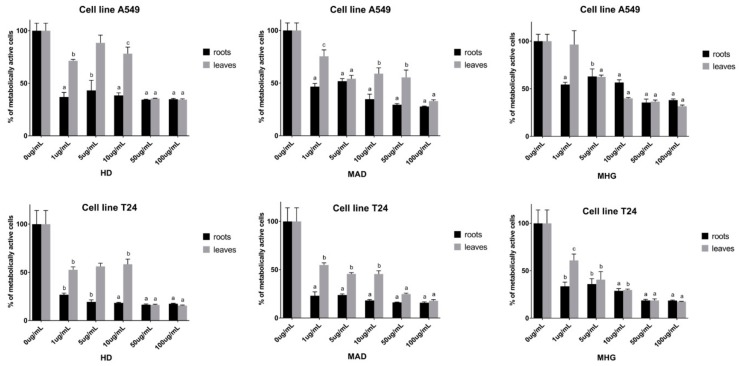
Percentage of metabolically active human lung carcinoma cell A549 and bladder cancer cell T24 lines after 72 h of incubation with different concentrations of volatiles obtained by HD, MAD, and MHG from the leaves and the roots of horseradish. Calculated IC_50_ values (μg/mL) are given in Table S1. Each data point is presented as mean ± SD (n = 3). Lower case letters represent significance level in comparison to non-treated cell line samples (a, *p* < 0.001; b, *p* < 0.01; c, *p* < 0.05).

**Figure 3:**
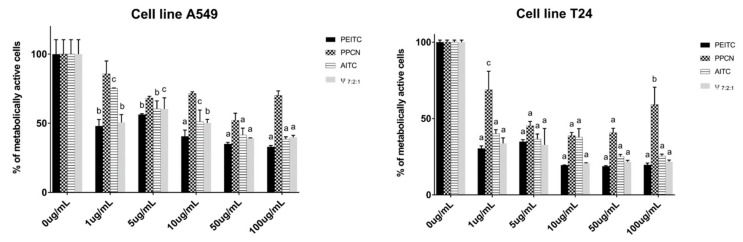
Percentage of metabolically active human lung carcinoma cell A549 and bladder cancer cell T24 lines after 72h of incubation with the main compounds obtained from horseradish isolates (2-phenylethyl ITC, 3-phenylpropanenitrile and allyl ITC) and their mixture (7:2:1, respectively). Calculated IC_50_ values (μg/mL) are given in Table S1. Each data point is presented as mean ± SD (n = 3). Lower case letters represent significance level in comparison to non-treated cell line samples (a, *p* < 0.001; b, *p* < 0.01; c, *p* < 0.05).

Cytotoxic activity of horseradish leaves and roots volatiles, individual compounds and their mixture was tested against human lung (A549) and bladder (T24) cancer cell lines. The results for tested concentrations after 72 h incubation period are shown in Figure 2 and Figure 3, while calculated IC_50_ values are given in Table S1. A549 was more resistant than T24 cell line, however, the results show that all horseradish volatiles obtained by different types of isolation have very high cytotoxic activity, which is consistent with the previous study [41].

The volatiles from the roots, in almost all cases, were more active than the ones from the leaves for both cell lines (Figure 2, Table S1). HD and MAD from the roots showed the highest activity on A549 having IC_50_ values of 2.62 and 4.08 μg/mL, respectively. In difference to the root volatiles which are represented by degradation products originating from 1 and 5, leaves volatiles are represented only by degradation products of 1. MHG from the leaves showed high cytotoxic activity, while MAD and HD showed moderate cytotoxic activity with IC_50_ values of 11.63, 23.47 and 34.22 μg/mL, respectively.

Even better activity was observed when testing leaves and roots volatile isolates against human bladder cancer cell line (T24). HD, MAD, MHG of all samples largely suppressed cell viability, with IC_50_ values of 0.57, 0.48, and 1.14 μg/mL, respectively for roots isolates and 7.87, 4.77, and 3.13 μg/mL, respectively for leaves isolates.

In order to investigate the contribution of the main constituents of the volatiles to the observed cytotoxic activity, PEITC, PPCN, and AITC were tested individually by MTT test (Figure 3, Table S1). PEITC showed the highest cytotoxic activity for both cell lines. When comparing the cytotoxic activity of PEITC, PPCN, and AITC against A549 cell line, PEITC and AITC showed high activity having IC_50_ values of 6.27 μg/mL and 17.76 μg/mL, respectively. On the other hand PPCN did not reach IC_50_ value. Thus it can be suggested that the observed root volatiles activities can be attributed to the high percentages of PEITC and AITC.

To investigate the contribution of AITC and PPCN to the PEITC activity, we tested the mixture of PEITC:PPCN:AITC in 7:2:1 ratio. This mixture having IC_50_ of 12.96 µg/mL was two times weaker than PEITC alone. In order to quantitatively determine interactions between the components a combination index (CI) method was used and calculated with CompuSyn software. The obtained CI value of 2.39 indicated antagonism of these three components.

PEITC showed the best activity against T24 cell line as well, with IC_50_ of 0.84 μg/mL, followed by AITC and PPCN, both showing high activity, with IC_50_ values of 1.96 μg/mL and 6.52 μg/mL, respectively. These results suggest that the observed high activities of the volatile isolates can be attributed to the presence of all three components. The roots volatile isolates, having PEITC as major constituent, showed better or very similar activity to PEITC indicating some type of interaction between its constituents (Figure 2 and Table S1). Mixture of PEITC:PPCN:AITC in 7:2:1 ratio was tested as well showing similar activity after 72 h incubation period (IC_50_ = 0.95 µg/mL) to PEITC itself. These observations indicate positive synergistic effect of the main constituents obtained from horseradish volatile isolates. CI value for T24 cell lines using the same mixture combination was calculated to be 0.61 which indicates synergism.

Tang et al. found that AITC and PEITC induce NAD(P)H:quinone oxidoreductase 1 and elevate glutathione levels in human bladder cancer cells. They also affect cell-cycle progression; AITC blocks cells in the G2/M phase while PEITC arrests cells in both G2/M and S phase. PEITC is more potent than AITC in the induction of apoptosis through the cellular activity of caspase 3/7 [45]. A study by Tripathi et al. showed that AITC induces replication stress-associated DNA damage response and slows cell cycle progression through S phase in human lung cancer cells [46], while another study by Kuang et al. discovered that PEITC induces apoptosis through elevated expression of P53 and P21 or necrosis, in concentration-dependent manner [47].

## 4. Conclusions

Different isolation techniques of horseradish roots and leaves isolates resulted in different yields of AITC, PEITC, and corresponding nitriles in the roots; whilst in the leaves, AITC was predominant regardless of the isolation method. Horseradish extract and distillates effectively inhibited a range of opportunistic pathogens, including the emerging food spoilage microorganisms as well as hospital multidrug-resistant ESKAPE strains. MHG proved to be the most active, inhibiting growth of MRSA, *L. monocytogenes*, clinical *A. baumannii* and fungi. The volatile susceptibility testing clearly showed that the antimicrobial activity of horseradish roots extract and distillates arises from the combined activity of its three main volatiles, among which the PEITC was the most active. PEITC was also shown to be the most contributing to the cytotoxic activity against two cell lines.

Over the past decade, intensive research has been devoted to ITCs as they are recognized to be responsible for various biological activities (anticancer, antimicrobial, etc.). Their activity is usually investigated using pure compounds or as a part of volatile mixtures isolated from plants that commonly contain 2-3 major GSLs as their precursors. On the other hand, the synergistic effects of their combinations are scarcely studied. Thus, in order to better understand the mechanisms involved in ITCs interactions, more detailed studies that comprise a higher number of ITCs and their different proportions in the mixtures are necessary.

## Figures and Tables

**Table 1:** Content of glucosinolates (μmol/g DW) in the roots and the leaves of horseradish.

Glucosinolate (Trivial Name)	*t*_R_ (min)	Roots (μmol/g DW)	Leaves (μmol/g DW)	[M + Na]^+^
***Aliphatic***				
**Allyl GSL (1)** * (Sinigrin)	2.0	3.53 ± 0.37	11.43 ± 0.26	302
**But-3-enyl GSL (2)** (Gluconapin)	4.4	tr	tr	316
**Pent-4-enyl GSL (3)** (Glucobrassicanapin)	6.0	tr	tr	330
***Branched***				
***sec*-Butyl GSL (4)** (Glucocochlearin)	5.4	tr	tr	318
***Arylaliphatic***				
**2-Phenylethyl GSL (5**) (Gluconasturtiin)	7.6	7.21 ± 0.25	tr	366
***Indole***				
**Indol-3-yl GSL (6)** (Glucobrassicin)	6.7	0.15 ± 0.08	tr	391
**Total** (μmol/g DW)		10.89 ± 0.70	11.43 ± 0.26	

[M + Na]^+^, sodium adduct of desulfoglucosinolate; DW, dry weight; tr, trace amounts (<0.1 μmol/g DW). * Data are presented as mean ± SD (n = 4). Numbers in parentheses correspond to structures given in Figure 1.

**Table 2:** Isothiocyanates and other volatiles identified in the roots and the leaves of horseradish by GC-MS.

Compound	RI_1_	RI_2_	HD		MAD		MHG	
			Roots	Leaves	Roots	Leaves	Roots	Leaves
But-3-enenitrile ^a,b,c^	1272	-	6.58	1.64	1.05	37.16	11.76	37.05
(*E*)-Hex-2-enal ^a,b,c^	1311	-	-	1.12	-	0.12	-	0.11
*sec*-Butyl isothiocyanate ^a,c^	1360	936	1.04	4.31	0.17	2.76	0.04	0.10
(*Z*)-Pent-2-en-1-ol ^a,b,c^	1393	-	-	0.07	-	-	-	0.06
Allyl isothiocyanate (AITC) ^a,b,c^	1429	879	46.36	73.45	14.29	54.77	13.81	52.36
(*Z*)-Hex-3-en-1-ol ^a,b,c^	1452	862	0.30	5.22	0.03	0.74	0.15	1.65
(*E*)-Hex-2-en-1-ol ^a,b,c^	1474	-	-	0.22	-	0.04	-	0.02
Nonanal ^a,b,c^	1481	-	-	-	-	0.41	-	-
Allyl thiocyanate ^a,c^	1504	-	1.15	1.76	0.32	0.41	0.26	0.31
But-3-enyl isothiocyanate ^a,b,c^	1514	992	0.51	0.20	0.10	0.08	0.04	0.04
Pent-4-enyl isothiocyanate ^a,c^	1589	1094	0.24	0.32	0.20	0.04	0.04	0.02
Benzeneacetaldehyde ^a,b,c^	1676	-	-	0.12	-	0.37	-	0.19
2-Methoxy-3-(1-methylpropyl)pyrazine ^a,b^	-	1173	0.37	-	0.10	-	0.06	-
2-Phenylethyl alcohol ^a,b,c^	1914	-	-	0.03	-	-	0.04	0.06
3-Phenylpropanenitrile (PPCN) ^a,b,c^	2024	1248	15.44	1.69	18.61	0.49	34.44	0.23
Octanoic acid ^a,b,c^	2056	-	0.01	0.15	0.24	0.25	-	0.00
Nonanoic acid ^a,b,c^	2154	-	0.01	-	0.15	0.12	0.70	0.12
(*E*)-*β*-Ionone ^a,b,c^	-	1493	-	-	-	1.28	-	-
2-Phenylethyl isothiocyanate (PEITC) ^a,b,c^	2197	1513	27.61	2.81	62.82	0.29	30.53	0.07
Decanoic acid ^a,b,c^	2254	-	0.01	0.96	0.15	0.12	0.83	0.77
Undecanoic acid ^a,b,c^	2351	-	-	0.57	0.10	0.04	0.44	0.41
Benzoic acid ^a,b,c^	2371	-	-	-	0.03	0.04	0.13	0.14
Tridecanoic acid ^a,b,c^	2561	-	-	0.33	-	-	0.13	0.14
Tetradecanoic acid ^a,b,c^	2645	-	-	0.74	-	-	0.35	0.33
Pentadecanoic acid ^a,b,c^	2744	-	-	0.50	-	-	0.46	0.40
**Total sum (%)**			99.66	96.18	98.36	99.55	94.22	94.60
**Yield (ng/g)**			66.34	138.96	35.43	3.96	7.57	3.39
Isothiocyanates (%)			75.76	81.09	77.58	57.94	44.46	52.59
Nitriles (%)			22.02	3.33	19.66	37.65	46.20	37.28
Others (%)			1.88	11.76	1.12	3.96	3.56	4.73

HD—hydrodistillation using Clevenger type apparatus; MAD—microwave-assisted distillation; MHG—microwave hydrodiffusion and gravity; Retention indices (RI_1_ and RI_2_) determined on a CP Wax 52 and VF-5MS capillary column, respectively; -, not detected; tr, traces (<0.01%) ^a^ Compound identified by mass spectra and RI comparison with homemade library; ^b^ Compound identified by mass spectra comparison with Wiley library; ^c^ Compound identified by mass spectra comparison with literature values [34,35,36].

**Table 3:** Antimicrobial activity of horseradish (*Armoracia rusticana*) roots extract, distillates by microdilution assay ^a^.

Species	Strain Origin	HD		MAD		MHG		Agent ^c^
		MIC	MBC ^b^	MIC	MBC	MIC	MBC	
**Gram-positive bacteria**								
*Listeria monocytogenes*	ATCC 19111	50	200	25	100	7.5	30	≤1 (S)
*Staphylococcus aureus*	ATCC 29213	25	>100	25	>100	30	>120	0.25 (S)
*Staphylococcus aureus*	Clinical/MRSA	50	>200	12.5	>50	3.75	>15	≥16 (R)
*Enterococcus faecalis*	ATCC 29212	50	200	50	200	15	60	≤1 (S)
*Streptococcus pyogenes*	ATCC 19615	50	200	50	200	15	60	≤1 (S)
*Bacillus cereus*	Food	50	50	25	25	15	15	≤1 (S)
**Gram-negative bacteria**								
*Salmonella* Typhimurium	WDCM 00031	50	100	25	50	15	30	≤1 (S)
*Escherichia coli*	ATCC 25922	50	50	25	50	15	30	0.5 (S)
*Escherichia coli*	Clinical	100	200	50	100	15	30	≤1 (S)
*Klebsiella pneumoniae*	ATCC 13883	100	200	100	100	30	30	0.12 (S)
*Klebsiella pneumoniae*	Clinical	200	400	100	100	30	30	≥16 (R)
*Acinetobacter baumannii*	ATCC 19606	50	100	25	50	7.5	15	1 (S)
*Acinetobacter baumannii*	Clinical	50	100	25	50	7.5	15	≥16 (R)
Fungi		MIC_50_	MIC_90_	MIC_50_	MIC_90_	MIC_50_	MIC_90_	MIC_90_
*Candida albicans*	Environmental	<0.78	≤0.78	<0.39	≤0.39	<0.12	≤0.12	1 (S)
*Penicillium notatum*	Food	3.125	6.25	3.125	6.25	0.47	0.94	0.5 (S)
*Aspergillus niger*	Food	3.125	6.25	1.56	3.125	0.47	0.94	0.5 (S)

^a^ All values are given in μg/mL. Stock solutions were made in 96% EtOH. EtOH was used as control. ^b^ MBC values were obtained by plating the aliquots from wells corresponding to MIC, 2 × MIC and 4 × MIC. ^c^ Gentamicin (10 μg) was used as standard for all bacterial strains, except for *L. monocytogenes* when erythromycin was used. Amphotericin B was tested against fungi. Abbreviations: S, susceptible; R, resistant.

**Table 4:** Antimicrobial activities of 2-phenylethyl ITC (PEITC) and 3-phenylpropanenitrile (PPCN), and mixed solutions of PEITC, PPCN and allyl ITC (AITC), in different ratios Ψ_PEITC:PPCN:AITC_ against tested strains by microdilution assay ^a^.

Species	Strain Origin	PEITC		PPCN		Ψ_7:2:1_^c^		Ψ_4:4:2_		Ψ_2:3:5_		Agent ^d^
		MIC	MBC ^b^	MIC	MBC	MIC	MBC	MIC	MBC	MIC	MBC	
**Gram-positive bacteria**												
*Listeria monocytogenes*	ATCC 19111	125	500	125	1000	12.5	50	7.5	30	25	100	≤1 (S)
*Staphylococcus aureus*	ATCC 29213	31.25	>125	500	2000	25	>100	30	>120	25	>100	0.25 (S)
*Staphylococcus aureus*	Clinical/MRSA	15.6	62.5	500	2000	25	50	7.5	30	50	100	≥16 (R)
*Enterococcus faecalis*	ATCC 29212	125	500	500	2000	12.5	12.5	15	60	50	200	≤1 (S)
*Streptococcus pyogenes*	ATCC 19615	62.5	250	500	2000	25	100	15	60	50	200	≤1 (S)
*Bacillus cereus*	Food	62.5	62.5	500	1000	25	25	15	15	50	50	≤1 (S)
**Gram-negative bacteria**												
*Salmonella* Typhimurium	WDCM 00031	62.5	125	500	1000	25	50	15	30	50	100	≤1 (S)
*Escherichia coli*	ATCC 25922	15.6	31.25	500	1000	25	25	30	60	50	50	0.5 (S)
*Escherichia coli*	Clinical	250	500	500	1000	50	100	15	30	100	200	≤1 (S)
*Klebsiella pneumoniae*	ATCC 13883	250	250	500	1000	50	100	30	30	100	200	0.12 (S)
*Klebsiella pneumoniae*	Clinical	500	500	500	1000	100	100	30	30	200	400	≥16 (R)
*Acinetobacter baumannii*	ATCC 19606	125	250	250	500	25	50	7.5	15	50	100	1 (S)
*Acinetobacter baumannii*	Clinical	31.25	31.25	125	500	12.5	25	7.5	15	25	50	≥16 (R)
**Fungi**		MIC_50_	MIC_90_	MIC_50_	MIC_90_	MIC_50_	MIC_90_	MIC_50_	MIC_90_	MIC_50_	MIC_90_	MIC_90_
*Candida albicans*	Environmental	<1.95	≤1.95	125	250	0.39	0.78	0.12	0.23	0.78	1.56	1 (S)
*Penicillium notatum*	Food	3.9	7.8	31.25	62.5	3.125	6.25	0.47	0.94	3.125	6.25	0.5 (S)
*Aspergillus niger*	Food	1.95	3.9	31.25	125	3.125	6.25	0.94	1.875	6.25	12.5	0.5 (S)

^a^ All values are given in μg/mL. Stock solutions were made in 96% EtOH. EtOH was used as control; ^b^ MBC values were obtained by plating the aliquots from wells corresponding to MIC, 2 × MIC and 4 × MIC; ^c^ Ψ_PEITC: PPCN:AITC,_ the ratio of the main volatiles found after different isolation methods PEITC, PPCN and AITC; ^d^ Gentamicin (10 μg) was used as standard for all bacterial strains, except for *L. monocytogenes* when erythromycin was used. Amphotericin B was tested against fungi. Abbreviations: S, susceptible; R, resistant.

## Supplementary Materials

The following are available online at https://www.mdpi.com/2218-273X/10/2/343/s1, Figure S1: Chromatogram of desulfoglucosinolates obtained from the roots and the leaves of horseradish: **d1**—desulfosinigrin; **d2**—desulfogluconapin; **d3**—desulfoglucobrassicanapin; **d4**—desulfoglucocochlearin; —desulfogluconasturtiin; **d6**—desulfoglucobrassicin, Figure S2: UV-Vis and MS^2^ spectra at 15V ionization of 3 main desulfoglucosinolates detected: **d1**, **d5**, and **d6**, Table S1: Calculated IC_50_ values (μg/mL) for volatiles obtained by HD, MAD and MHG from the roots and the leaves of horseradish and its main compounds 2-phenylethyl ITC, 3-phenylpropanenitrile, allyl ITC, and their mixture in the proportion similar to the one obtained by root MAD, 7:2:1, respectively (Ψ_7:2:1_) against human lung cancer cell A549 and bladder cancer cell T24 lines after 72 h.
