# Supplementary material for: Biological Effects of Glucosinolate Degradation Products from Horseradish: A Horse that Wins the Race
Source: Biomolecules. 2020 Feb 21;10(2):343. doi: 10.3390/biom10020343 (PMC7072351; doi:10.3390/biom10020343)
Supplement: Supplementary file 1 [file biomolecules-10-00343-s001.zip › Supplementary Materials_blazevic_proof.docx]

**Supplementary Materials**

Article

**Biological effects of glucosinolate degradation products from horseradish: A horse that wins the race**

**Marijana Popović ^1^*, Ana Maravić ^2^, Vedrana Čikeš Čulić ^3^, Azra Đulović ^1^, Franko Burčul ^4^, Ivica Blažević ^1^***

Department of Organic Chemistry, Faculty of Chemistry and Technology, University of Split, Ruđera Boškovića 35, Split 21000, Croatia; azra@ktf-split.hr

^2^ Department of Biology, Faculty of Science, University of Split, Ruđera Boškovića 33, Split 21000, Croatia; amaravic@pmfst.hr

^3^ Department of Medical Chemistry and Biochemistry, School of Medicine, University of Split, Šoltanska 2, Split 2100, Croatia; vcikesc@mefst.hr

^4^ Department of Analytical Chemistry,Faculty of Chemistry and Technology, University of Split, Ruđera Boškovića 35, Split 21000, Croatia; franko@ktf-split.hr

***** Correspondence: [mpopovic@ktf-split.hr](mailto:mpopovic@ktf-split.hr) (M.P.); [blazevic@ktf-split.hr](mailto:blazevic@ktf-split.hr) (I.B.); Tel.:+385 21 329 434, Ivica Blažević

Received: date; Accepted: date; Published: date

**d3**

**d4d**

**d20**

**d6**

**d1**

**d5**

**d6**

**d3**

**d4**

**d2**

**d5**

**d1**

**Figure S1.** Chromatogram of desulfoglucosinolates obtained from the roots and the leaves of horseradish: **d1** - desulfosinigrin; **d2** - desulfogluconapin; **d3** - desulfoglucobrassicanapin; **d4** - desulfoglucocochlearin; **d5** – desulfogluconasturtiin; **d6** - desulfoglucobrassicin.

**d1**

**d1**

**d5**

**d5**

**Figure S2.** UV-Vis and MS^2^ spectra at 15V ionization of 3 main desulfoglucosinolates detected: **d1**, **d5**, and **d6**.

**d6**

**d6**

**Table S1.** Calculated IC_50_ values (μg/mL) for volatiles obtained by HD, MAD and MHG from the roots and the leaves of horseradish and its main compounds 2-phenylethyl ITC, 3-phenylpropanenitrile, allyl ITC, and their mixture in the proportion similar to the one obtained by root MAD, 7:2:1, respectively (**Ѱ_7:2:1_**) against human lung cancer cell A549 and bladder cancer cell T24 lines after 72h.

| **Cell line** | **HD** | | **MAD** | | **MHG** | | **PEITC** | **PPCN** | **AITC** | **Ѱ_7:2:1_** |
| --- | --- | --- | --- | --- | --- | --- | --- | --- | --- | --- |
|  | **roots** | **leaves** | **roots** | **leaves** | **roots** | **leaves** |  |  |  |  |
| **A549** | 2.62 | 34.22 | 4.08 | 23.47 | 14.34 | 11.63 | 6.27 | >100 | 17.76 | 12.96 |
| **T24** | 0.57 | 7.87 | 0.48 | 4.77 | 1.14 | 3.13 | 0.84 | 6.52 | 1.96 | 0.95 |

HD – hydrodistillation in Clevenger type apparatus; MAD - microwave-assisted distillation; MHG - microwave hydrodiffusion and gravity; PEITC - 2-phenylethyl isothiocyanate; PPCN -3-phenylpropanenitrile; AITC- allyl isothiocyanate.
