# Supplementary material for: Biological Effects of Glucosinolate Degradation Products from Horseradish: A Horse that Wins the Race
Source: Biomolecules. 2020 Feb 21;10(2):343. doi: 10.3390/biom10020343 (PMC7072351; doi:10.3390/biom10020343)
Supplement: Supplementary file 1 [file biomolecules-10-00343-s001.zip › Supplementary Materials_blazevic_proof.pdf]

biomolecules

Supplementary Materials

Article

Biological effects of glucosinolate degradation products from horseradish: A horse that wins the race

Marijana Popović [1]*, Ana Maravić [2], Vedrana Čikeš Čulić [3], Azra Đulović [1], Franko Burčul [4], Ivica Blažević [1]*

Department of Organic Chemistry, Faculty of Chemistry and Technology, University of Split, Ruđera Boškovića 35, Split 21000, Croatia; azra@ktf-split.hr
[2] Department of Biology, Faculty of Science, University of Split, Ruđera Boškovića 33, Split 21000, Croatia; amaravic@pmfst.hr
[3] Department of Medical Chemistry and Biochemistry, School of Medicine, University of Split, Šoltanska 2, Split 2100, Croatia; vcikesc@mefst.hr
[4] Department of Analytical Chemistry,Faculty of Chemistry and Technology, University of Split, Ruđera Boškovića 35, Split 21000, Croatia; franko@ktf-split.hr
* Correspondence: mpopovic@ktf-split.hr (M.P.); blazevic@ktf-split.hr (I.B.); Tel.:+385 21 329 434, Ivica Blažević

Received: date; Accepted: date; Published: date

biomolecules

MDPI

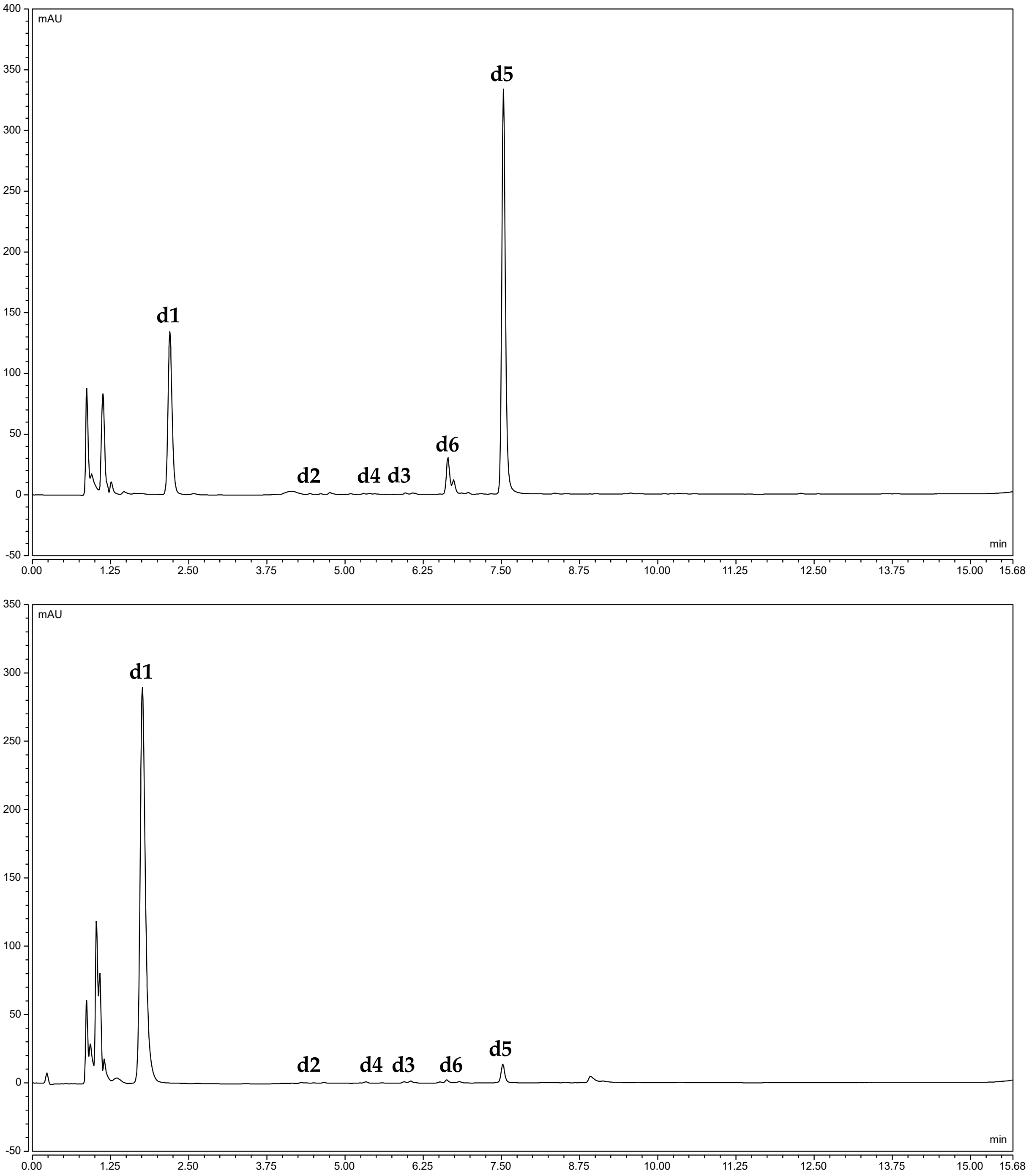

Figure S1. Chromatogram of desulfoglucosinolates obtained from the roots and the leaves of horseradish: **d1** - desulfosinigrin; **d2** - desulfogluconapin; **d3** - desulfoglucobrassicanapin; **d4** - desulfoglucocochlearin; **d5** – desulfogluconasturtiin; **d6** - desulfoglucobrassicin.

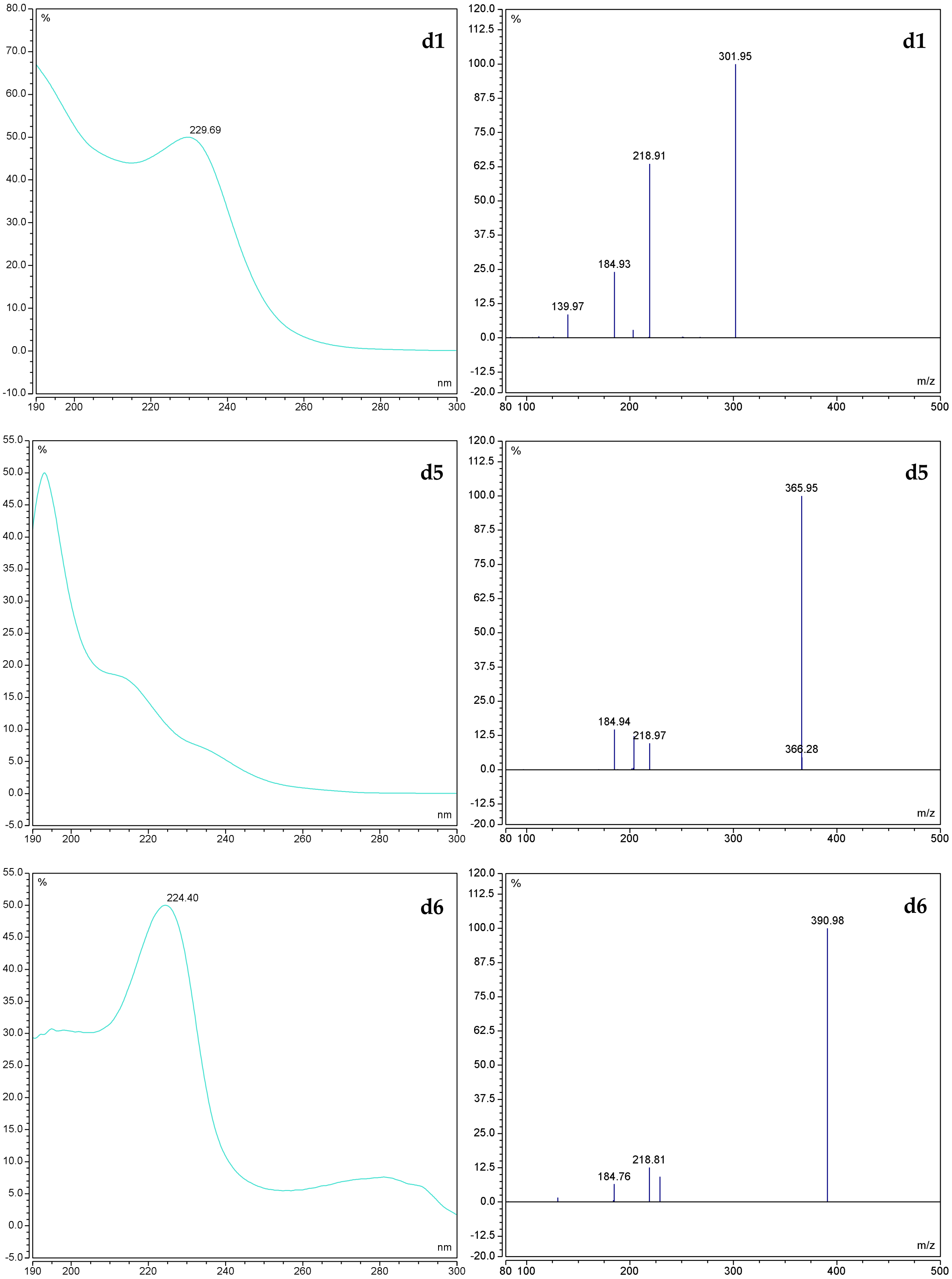

Figure S2. UV-Vis and MS² spectra at 15V ionization of 3 main desulfoglucosinolates detected: **d1**, **d5**, and **d6**.

biomolecules

MDPI

Table S1. Calculated IC_{50} values (μg/mL) for volatiles obtained by HD, MAD and MHG from the roots and the leaves of horseradish and its main compounds 2-phenylethyl ITC, 3-phenylpropanenitrile, allyl ITC, and their mixture in the proportion similar to the one obtained by root MAD, 7:2:1, respectively ($\Psi_{7:2:1}$) against human lung cancer cell A549 and bladder cancer cell T24 lines after 72h.

Cell line	HD		MAD		MHG		PEITC	PPCN	AITC	$\Psi_{7:2:1}$
	roots	leaves	roots	leaves	roots	leaves				
**A549**	2.62	34.22	4.08	23.47	14.34	11.63	6.27	>100	17.76	12.96
**T24**	0.57	7.87	0.48	4.77	1.14	3.13	0.84	6.52	1.96	0.95

HD – hydrodistillation in Clevenger type apparatus; MAD - microwave-assisted distillation; MHG - microwave hydrodiffusion and gravity; PEITC - 2-phenylethyl isothiocyanate; PPCN -3-phenylpropanenitrile; AITC- allyl isothiocyanate.
